# Unpacking the effects of personality traits on algorithmic awareness: The mediating role of previous knowledge and moderating role of internet use

**DOI:** 10.3389/fpsyg.2022.953892

**Published:** 2022-09-06

**Authors:** Wei Fang, Jianbin Jin

**Affiliations:** ^1^School of Public Administration and Communication, Beijing Information Science and Technology University, Beijing, China; ^2^School of Journalism and Communication, Tsinghua University, Beijing, China

**Keywords:** big five, personality traits, algorithm, awareness, previous knowledge, mediating role, moderating role, internet use

## Abstract

The COVID-19 pandemic has accelerated the integration of algorithms in online platforms to facilitate people’s work and life. Algorithms are increasingly being utilized to tailor the selection and presentation of online content. Users’ awareness of algorithmic curation influences their ability to properly calibrate their reception of online content and interact with it accordingly. However, there has been a lack of research exploring the factors that contribute to users’ algorithmic awareness, especially in the roles of personality traits. In this study, we explore the influence of Big Five personality traits on internet users’ algorithmic awareness of online content and examine the mediating effect of previous knowledge and moderating effect of breadth of internet use in in China during the pandemic era. We adapted the 13-item Algorithmic Media Content Awareness Scale (AMCA-scale) to survey users’ algorithmic awareness of online content in four dimensions. Our data were collected using a survey of a random sample of internet users in China (*n* = 885). The results of this study supported the moderated mediation model of open-mindedness, previous knowledge, breadth of internet use, and algorithmic awareness. The breadth of internet use was found to be a negative moderator between previous knowledge and algorithmic awareness.

## Introduction

Recently, the Coronavirus disease (COVID-19) pandemic has deepened our dependency on the internet and accelerated the integration of algorithms in our daily lives. The number of internet users has increased greatly during the pandemic. According to the International Telecommunication Union (ITU), the number of internet users grew from 4.1 billion in 2019 to 4.9 billion (or 54% of the world’s population) in 2021 (ITU, 2021). Furthermore, internet users in China are spending more time online for news consumption, information acquisition, social interaction, entertainment, etc. (CNNIC, 2020). Meantime, we are increasingly living in a society that is governed and structured by algorithms, which are broadly defined as encoded procedures driven by specified calculations, which transform input data into a desired output (Gillespie, 2014). In the algorithm-driven online environment, social media and other types of platforms decide what flow of content we see, determine what ads we would like, make content recommendations that cater to our needs, and moderate content by detecting and removing content that is deemed inappropriate (Rainie et al., 2022).

The increasing integration of algorithms among online platforms have stimulated studies examining issues surrounding users’ algorithmic awareness. Algorithmic awareness mainly refers to whether individuals are aware of the operations and impact of algorithms in their everyday life. The existing studies relating to algorithmic awareness have centered on two different perspectives, namely, the specific and general perspectives. Researchers holding the specific perspective tend to define and measure algorithmic awareness based on whether users have awareness of the roles that algorithms play in particular platforms, e.g., Facebook and Google (Rader, 2014; Eslami et al., 2015; Rader and Gray, 2015). On the other hand, scholars also argue that in a society with an increasing utilization of algorithms in almost every aspect of individuals’ lives, being aware of specific algorithms used by particular platforms demonstrates more than enough awareness (Eslami et al., 2015). Hence algorithmic awareness is defined within a broader level and is often interwoven with discussions on experiences, privacy concerns, normative values, and literacy surrounding algorithms. Cotter and Reisdorf (2020) focus on search engines and define algorithmic awareness as the extent to which internet users are aware of why and how algorithms are used to prioritized certain information in their online search results and the potential impact of algorithms on them and certain social groups. In addition to special type of platforms, studies have also been conducted to examine whether users are aware of algorithms in different domains of their daily life. In a qualitative interview study conducted among 30 German internet users, the authors examined users’ awareness of algorithmic operations in eight domains of internet use, and found that the participants were well aware of algorithm use in areas of advertising, online shopping, and streaming media use, whereas they had little awareness of algorithm use in the domains of news selection, navigation systems, jobs and dating services (Dogruel et al., 2020).

Furthermore, results of several empirical studies have shown a relatively low level of awareness of algorithms among users, despite the increasing usage of algorithms. From a more general perspective, the results of population surveys among internet users demonstrate that users’ awareness of the dynamics of algorithm usage is limited (Grzymek and Puntschuh, 2019; Gran et al., 2021). Most European internet users do not know what an algorithm is, even though they are widely used in many everyday online applications. In Norway, which is a country with nearly universal internet adoption, approximately 60% of users reported having low or no awareness of algorithms (Gran et al., 2021). From a more specific perspective, research focused on particular types of algorithms on specific online platforms has provided additional insights into users’ awareness of the algorithms they encounter. For widely adopted social media such as Facebook, most respondents (60%) are not aware of Facebook’s use of algorithms to filter their news feed (Eslami et al., 2015). In other words, they believe that they can see all the posts that their friends create on Facebook. Although participants from another study displayed a higher level of awareness (73%) of Facebook’s algorithm use, this was more likely representative of the increased level of awareness among more skilled internet users, as this study was conducted among Amazon’s Mechanical Turk users (Rader and Gray, 2015).

Considering the impact of algorithm-driven platforms on shaping user’ online environment and their low level of algorithmic awareness, it is crucial to investigate factors that can predict users’ awareness of algorithms. At the individual level, being aware of the operations and impact of algorithms is essential for users to protect their privacy (Rader, 2014), increase their level of autonomy online (Dogruel et al., 2020), diversify the sources of their information and increase their income, which are dependent on acquiring online attention (Klawitter and Hargittai, 2018; Bishop, 2019). Furthermore, on the societal level, users’ increasing level of awareness could also contribute to a more fair and transparent design of algorithms (Shin et al., 2022a), and thereby offer potentials to ensure algorithms as a tool for social justice. Thereby, this study aims to examine the factors that influence users’ algorithmic awareness in China during the pandemic era. More specifically, the purpose of this study is to examine the relationships between the Big Five personality factors, algorithmic awareness, previous knowledge, and breadth of internet use.

## Literature review

### Personality traits and algorithmic awareness

Personality traits are generally defined as relatively stable patterns of feelings, thoughts, and behaviors reflected by individuals’ tendencies to react under certain circumstances (McElroy et al., 2007; Roberts, 2009). The Big Five framework consists of five subscales of personality traits, namely, agreeableness, extraversion, conscientiousness, neuroticism, and openness to experience. Each dimension of the Big Five personality framework represents a domain of unique personality traits. Individuals with a high level of openness tend to be more curious and seek new experiences; more conscientious individuals are more likely to be well organized, responsible, and reliable; more extraverted individuals tend to be more energetic, assertive, and excitement seeking; more agreeable individuals are more likely to be friendly, warm, and helpful; and those with a high level of neuroticism tend to be more anxious and moodier (Sharan and Romano, 2020). The Big Five personality traits have been widely used for describing personal characteristics in the last three decades (Zhang et al., 2021) and have been shown useful in explaining individual differences in social attitudes and behaviors (McCrae, 2000).

The increasing integration of artificial intelligence (AI) in many domains of society has also stimulated studies to examine the relationships between personality traits and AI. Personality traits have been shown to play a moderating role in the acceptance of medical AI, with openness and conscientiousness strengthening the relations between human-computer trust and acceptance of AI for independent diagnosis and treatment and conscientiousness and agreeableness lessening the association between human-computer trust and acceptance of AI for assistive diagnosis and treatment (Huo et al., 2022). Furthermore, the Big Five personality traits have also been demonstrated to have an impact on human-AI trust (Sharan and Romano, 2020). More agreeable and conscientious people are more likely to have a higher level of trust in automation, according to a study conducted among participants from America, Taiwan, and Turkey (Chien et al., 2016). In addition to trust in automation, people’s trust in virtual reality (VR) teams has been found to relate to the Big Five personality traits, with agreeableness and extraversion negatively predicting trusting behavior and openness negatively relating to technology anxiety (Jacques et al., 2009). In addition to acceptance and trust in AI, personality traits have also been demonstrated to be associated with attitudes toward autonomous vehicles (AVs); more conscientious individuals tend to have greater concern with AVs, those with a high level of emotional stability and openness seem to be more eager to adopt AVs, more conscientious individuals are less likely to adopt AVs, more open individuals tend to be more willing to relinquish driving control, and more extroverted individuals are less likely to relinquish such control (Charness et al., 2018).

Recently, in addition to the above areas mentioned, algorithm technologies have been increasingly integrated into many of the popular internet platforms. This new type of technology has played a crucial role in automatically determining what content users encounter in the online environment. However, there has been a lack of research examining the roles of personality traits in the usage of algorithm technology in online platforms. Although some scholars have proposed integrating personality traits into an educational recommender system, drawing on the correlation between personality traits and academic performance demonstrated by previous studies (Gianotti et al., 2019), there is still a lack of empirical study examining the relationship between personality traits and algorithmic awareness and other related issues. However, the above-mentioned development in research on AI and personality traits and the recent theoretical development on the awareness of algorithms seem to provide new opportunities to integrate these two lines of research. In the area of algorithmic awareness, building on previous conceptualization of algorithms as experience technologies (DeVito et al., 2018; Cotter and Reisdorf, 2020), Swart (2021) defines algorithmic awareness as users’ understanding of the presence and operations of algorithms in the context of news selection on social media. Building on previous discussions on fairness, accountability, and transparency of algorithmic awareness, Shin (2021a) constructs awareness with four subscales: fairness, accountability, transparency, and explainability (FATE) Shin and Park (2019), Shin (2021b), Shin et al. (2022a) and proposes that FATE can be extended as a basis of algorithmic literacy (Shin et al., 2021c; Shin, 2022b). Specifically, in the area of algorithmic content recommendations, Zarouali et al. (2021) constructs the framework of algorithmic media content awareness (AMCA) in four dimensions, including users’ awareness of content filtering, automated decision-making, human-algorithm interplay, and ethical considerations. Furthermore, the ACMA framework, as proposed by the authors, can be seen as a theoretical concept used for examining how variables (e.g., media use. tech savviness, etc.) predict variations in awareness.

Drawing on the studies presented above surrounding the relationship between personality traits and AI, as well the development of theoretical framework in algorithmic awareness, we employ AMCA to measure users’ awareness of how algorithm operations as well as theoretical framework to explore predicting factors of algorithmic awareness in this study. We propose our first research question as follows:

RQ1: Are there correlations between certain dimensions of the Big Five personality traits and algorithmic awareness?

Besides, previous studies have demonstrated that demographic factors are predictors of users’ awareness of the use of algorithms in online applications. A recent national survey in Norway has shown strong associations between age, gender, education, geographic location and awareness of algorithms (Gran et al., 2021). More specifically, those who are older, less educated, female, and living outside the most populated urban areas seem to have a lower level of algorithmic awareness compared to those who are younger, well educated, male, and live within urban areas. Moreover, those whose occupations require higher level of engagement with several specific algorithm-driven platforms tend to have higher level of awareness (Klawitter and Hargittai, 2018). Thus, drawing on results of these studies, we include variables of age, gender, and education as control variables of this study.

### Mediating role of previous knowledge in algorithmic awareness

Internet users’ previous knowledge about algorithms contains all they have heard of or learned regarding algorithm technologies. This knowledge can be either general, for instance, knowing that there is a new technology called an algorithm that has been increasingly influencing the information we encounter in our daily lives (Hargittai et al., 2020), or very specific, for instance, knowing that social media platforms such as Facebook use algorithms to filter users’ news feed based on their online behaviors (Rader, 2014; Rader and Gray, 2015).

On the one hand, the acquisition of users’ previous knowledge of algorithms is associated with several factors. First, demographic factors have been demonstrated to contribute to users’ acquisition of algorithm-related knowledge. Similar to previous knowledge gap studies, males, young individuals, and well-educated individuals are more likely to have a higher level of knowledge regarding algorithms (Grzymek and Puntschuh, 2019; Cotter and Reisdorf, 2020). Second, algorithmic knowledge can also be obtained from other sources. Users can learn about algorithms through reading media reports on the related topics, gaining information through interpersonal networks, or professional training courses for those graduating or working in related areas (DeVito et al., 2018; Bishop, 2019; Cotter, 2019). Moreover, personality traits may also be able to facilitate users’ practices of accumulating knowledge of algorithms, through increasing their level of technology acceptance and usage (Devaraj et al., 2008), increasing their time spent online (Swickert et al., 2002; Landers and Lounsbury, 2006), though sometimes this could lead to certain problematic use (Kayiş et al., 2016; Zhou et al., 2018).

On the other hand, previous knowledge can contribute to one’s awareness of algorithms. Users’ knowledge of algorithms reminds them that a new technology called an algorithm has been widely used in online platforms, although many do not know whether their frequently used applications have used this technology or how exactly the technology operates. Previous studies on users’ awareness of algorithms, although they do not aim to examine the mediating role of previous knowledge between personality characteristics and algorithmic awareness, have provided evidence on the predictors of gaining algorithm knowledge and the impact of previous knowledge on increasing awareness (Grzymek and Puntschuh, 2019). In particular, those whose income is highly dependent on increasing the online visibility of their content, which is manipulated by algorithms, are more motivated to integrate their previous knowledge while on specific platforms and to exert more effort toward increasing their awareness of algorithms in arranging online content (Klawitter and Hargittai, 2018).

Therefore, it is reasonable to speculate that users’ previous knowledge about algorithms lays the foundation for their awareness of how algorithms operate either on specific platforms or in certain domains of internet use. Although there has been a lack of research examining the relationship between users’ previous knowledge and algorithmic awareness, implications from several studies seem to provide insights into this issue. Thus, drawing on the above discussions, we propose our second research question as follows:

RQ2: Is there a mediation effect of previous knowledge between certain dimensions of the Big Five personality traits and algorithmic awareness?

### Moderating role of internet use in previous knowledge and algorithmic awareness

It is worth noting that passive exposure to the algorithms alone does not guarantee users’ awareness of the algorithms’ use. Algorithms are experience technologies that cannot be easily understood without firsthand usage (Blank and Dutton, 2012). A study among Facebook users indicated that being a regular user for many years, having a relatively large network size, and having a certain number of stories is not associated with users’ awareness of Facebook’s News Feed algorithm (Eslami et al., 2015). Instead, users who use Facebook frequently, post stories actively, and adjust their settings frequently are more likely to be aware of Facebook’s algorithm’s function in content filtering (Eslami et al., 2015). Thus, the extent of the use of algorithm-driven platforms could play certain roles between individuals’ accumulation of knowledge and awareness of algorithms. For individuals with intensive use of online platforms, their usage would enrich their general knowledge about algorithms and contribute to the increase of their awareness of how algorithms works and the impact on their daily life (Blank and Dutton, 2012).

There have been evidences indicating that the level of breadth and depth of internet use are correlated with users’ experiences of accumulating knowledge and increasing algorithmic awareness. The breadth of internet use refers to the diversity of internet use (e.g., online activities, online applications), whereas the depth of internet use refers to the extent of users’ engagement online. Users who use Facebook more frequently tend to correlate with more knowledge and greater awareness of the platform’s news feed ranking algorithms and its working process (Eslami et al., 2015; Rader and Gray, 2015). The frequency and breadth of internet search on search engines are positively related with their knowledge and understanding about algorithms in online search (Cotter and Reisdorf, 2020). Online entrepreneurs and content creators, who are more motivated to engage deeply with specific platforms to promote their products or content, their online experiences relate to their knowledge and awareness of algorithms (Klawitter and Hargittai, 2018; Cotter, 2019). Drawing on the above discussions, we propose our third research question as follows:

RQ3: Is there a moderating effect of the breadth of internet use between previous knowledge and algorithmic awareness?

## Materials and methods

### Participants and procedures

The participants were recruited online using mixed methods. The first method used was an online crowdsourcing platform in mainland China (wjx), which provides functions equivalent to Amazon Mechanical Turk. Participants recruited through this platform (560) tend to be young and well educated, and most are between 20 and 40 years old and have a college degree. Furthermore, we used China’s popular social media platform WeChat to recruit participants. The demographic features of participants who are recruited on this social media platform (301) tend to be more diverse, covering age groups ranging from 10 to 19 years old to 60 years old and above and education levels ranging from primary and secondary to a college degree. Each respondent from WeChat groups received 5-10 Chinese yuan for his or her participation, which was paid by WeChat’s red packet function. In addition, as the link to the questionnaire was also available on an app targeting young internet users from across the whole country, we also received a small group of respondents (24) from the users of this platform.

The demographic characteristics of the participants are shown in Table 1. Among the participants, 43.5% were male, and 56.5% were female. More than half of the participants were from the 20–29 (34.1%) and 30–39 (35.3%) age groups, followed by the 40–49 (13.4%), 50–59 (8.7%), 10–19 (4.6%), and 60 and above (3.8%) age groups. Regarding the level of education, one-third of the participants had an undergraduate degree or above (70.2%), followed by a vocational college degree (18.2%), senior secondary level (8.1%), junior secondary level (3.1%), and primary level (0.5%). Considering that the Big Five personality scale adopted in this research consists of 60 complicated questions that require participants with a relatively higher level of education, the high percentage of participants with undergraduate and above degrees was crucial in ensuring the quality of personality trait-related data.

**TABLE 1:** Demographic profiles.

Demographic Variable	Item	Frequency	Percentage (%)	Cumulative percentage (%)
Gender	Male	385	43.5	43.5
	Female	500	56.5	100.0
Age	10–19	41	4.6	4.6
	20–29	302	34.1	38.8
	30–39	312	35.3	74.0
	40–49	119	13.4	87.5
	50–59	77	8.7	96.2
	≥ 60	34	3.8	100.0
Education	Primary	4	0.5	0.5
	Junior secondary	27	3.1	3.5
	Senior secondary	72	8.1	11.6
	Vocational college	161	18.2	29.8
	Undergraduate and above	621	70.2	100.0
Total		885	100.0	100.0

### Measures

#### Previous knowledge of algorithm technology

Building on previous research on users’ experiences with AI and practices of knowledge accumulation surrounding AI and algorithms (Charness et al., 2018), we included the variable ‘Users’ previous knowledge of algorithm technology’. Participants’ previous knowledge was measured by their self-reported level of knowledge about algorithm technology from a more general perspective. More specifically, the question of ‘Have you heard about algorithm technology and its usage in many popular online platforms?’ was presented to the participants. The participants rated their level of previous knowledge about his question using a 5-point scale (1 = not at all aware, 5 = completely aware) (M = 3.32, SD = 0.850).

#### Big five personality traits

We assessed participants’ personality traits using the Chinese version of the Big Five Inventory-2 (BFI-2) (Zhang et al., 2021), which has been tested to show good reliability, structural validity, convergent/discriminant validity, and criterion-related validity at the domain level. The BFI-2 consists of a total of 60 items nested within 5 12-item domains. Each of the items start with ‘I am someone who’ and end with a personality characteristic (e.g., ‘I am someone who: is outgoing, sociable’). Participants rated themselves on each item using a 5-point scale (1 = disagree strongly, 5 = agree strongly). The survey produced a score for each of the Big Five personality traits for respondents: extraversion (*M* = 3.259, SD = 1.017, α = 0.850), agreeableness (*M* = 3.936, SD = 0.741, α = 0.848), conscientiousness (*M* = 3.823, SD = 0.883, α = 0.877), negative emotionality (*M* = 2.472, SD = 0.963, α = 0.886), open-mindedness (*M* = 3.493, SD = 0.935, α = 0.856).

#### Breadth of internet use

Building on previous studies on internet users’ online experiences and engagement with algorithms (Eslami et al., 2015; Cotter and Reisdorf, 2020), we included the variable ‘the breadth of users’ internet use’. Respondents’ breadth of internet use was measured by their self-reported level of the breadth of online applications usage. More specifically, the question of ‘How many online applications do you use frequently in your daily life?’ was presented to the respondents. The respondents rated their level of the breadth of internet use using a 5-point scale (1 = less than 10, 5 = 40 and above) (*M* = 1.73, SD = 0.792).

#### Algorithmic awareness of online content

We adopted the AMCA-scale, developed by Zarouali et al. (2021) to measure Chinese users’ awareness of the algorithm’s role in selecting and presenting online content. The AMCA-scale is designed to measure internet users’ ‘awareness of the usage and consequences of algorithms for the media content’ on online platforms such as Netflix, YouTube, and Facebook (Zarouali et al., 2021, p. 9). The scale consists of 13 items that specifically measure users’ level of awareness regarding the usage of algorithms in content filtering, automated decision-making, human-algorithm interplay, and the related ethical considerations. Each of the items is a statement indicating the role of algorithms in media content (e.g., Algorithms are used to recommend online content to me). Participants rate themselves on each statement using a 5-point scale (1 = not at all aware, 5 = completely aware) (*M* = 3.539, SD = 1.033, α = 0.913).

### Statistical analysis

In this study, we utilized SPSS (version 26, IBM Corp.) and PROCESS (version 4.0, Hayes) software applications to examine the effect of the mediating and moderating factors. We first present descriptive statistics for our control variables and variables of interest, followed by bivariate associations among these variables. Second, we presented the results of regression analysis of the dependent variable. Third, we used PROCESS to examine the mediating effect of previous knowledge between open-mindedness and algorithmic awareness, and the moderating effect of the breadth of internet use between previous knowledge and algorithmic awareness.

## Results

### Descriptive statistics and correlation analysis

The descriptive statistics of and correlations between the study variables are presented in Table 2. There were positive correlations between extraversion and algorithmic awareness (*r* = 0.270, *p* < 0.01), agreeableness and algorithmic awareness (*r* = 0.221, *p* < 0.01), conscientiousness and algorithmic awareness (*r* = 0.211, *p* < 0.01), and open-mindedness and algorithmic awareness (*r* = 0.357, *p* < 0.01), whereas there was a negative correlation between negative emotionality and algorithmic awareness (*r* = –0.228, *p* < 0.01). Similarly, extraversion, agreeableness, conscientiousness, and open-mindedness had a positive correlation with previous knowledge (*r* = 0.246 ∼ 0.361, *ps* < 0.01), whereas negative emotionality had a negative correlation with previous knowledge (*r* = –0.231, *p* < 0.01). Previous knowledge was positively correlated with algorithmic awareness (*r* = 0.568, *p* < 0.01). The breadth of internet use was positively correlated with previous knowledge (*r* = 0.218, *p* < 0.01) and algorithmic awareness (*r* = 0.183, *p* < 0.01).

**TABLE 2:** Descriptive statistics and correlation analyses for each research variable.

	1	2	3	4	5	6	7	8	9	10	11
Open-Mindedness	–										
Negative Emotionality	–0.520**	–									
Conscientiousness	0.515**	–0.704**	–								
Agreeableness	0.454**	–0.615**	0.675**	–							
Extraversion	0.608**	–0.678**	0.606**	0.469**	–						
Previous Knowledge	0.361**	–0.231**	0.246**	0.276**	0.306**	–					
Breadth of Internet Use	0.199**	–0.178**	0.146**	0.149**	0.224**	0.218**	–				
Algorithmic Awareness	0.357**	–0.228**	0.211**	0.221**	0.270**	0.568**	0.183**	–			
Gender	0.076*	–0.178**	0.107**	–0.018	0.133**	0.076*	0.082*	0.071*	–		
Age	–0.089**	–0.063	0.112**	–0.033	0.004	–0.177**	–0.101**	–0.242**	0.051	–	
Edu	0.195**	–0.107**	0.138**	0.153**	0.120**	0.238**	0.136**	0.327**	–0.009	–0.288**	–
M	3.49	2.47	3.82	3.94	3.30	3.32	1.73	3.54	1.44	3.99	4.55
SD	0.60	0.65	0.61	0.53	0.63	0.85	0.79	0.71	0.50	1.16	0.81

Sample size = 885; * *p* < 0.05 **; *p* < 0.01.

### Control variables

Gender and level of education had positive effects on users’ algorithmic awareness, whereas age had a negative effect on algorithmic awareness. More specifically, male participants (*β* = 0.082, *p* < 0.01) and those with a higher level of education (*β* = 0.280, *p* < 0.001) tended to be more aware of the roles of the algorithm in the content they encountered online; in addition, with an increase in age, the level of algorithmic awareness decreased (*β* = –0.165, *p* < 0.001).

### Regression analysis

**FIGURE 1:**
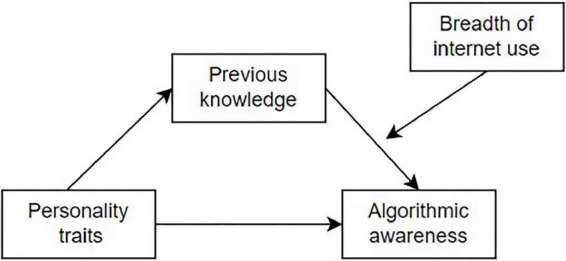
The theoretical model.

Table 3 shows the results of the regression analysis of algorithmic awareness as the dependent variable. First, the control variables were included in the regression, and then five dimensions of Big Five personality traits were included in the regression model (Models 1 and 2). The third step was to add previous knowledge to the regression model (Model 3). The fourth step added breadth of internet use and the interaction item to the regression model (Model 4). The results of model 2 indicated that among the five dimensions of Big Five personality traits, only the dimension of open-mindedness was significantly correlated with the dependent variable. Thereby, the first research question is answered: open-mindedness is positively correlated with algorithmic awareness. Furthermore, the results presented in model 3 and 4 have demonstrated the correlations between previous knowledge, the breadth of internet use with algorithmic awareness. Drawing on these results, we will analyze the mediating effect of previous knowledge between open-mindedness and algorithmic awareness, and the moderating effect of breadth of internet use between previous knowledge and algorithmic awareness. We propose the theoretical model shown in Figure 1.

**TABLE 3:** Hierarchical regression results for the level of algorithmic awareness.

Variable	Dependent variable: Level of algorithmic awareness			
	Model 1	Model 2	Model 3	Model 4
Constant	2.649***	1.608***	1.596***	1.031*
Gender	0.082**	0.052	0.023	0.026
Age	–0.165***	– 0.160***	– 0.108***	– 0.102***
Education	0.280***	0.221***	0.160***	0.154***
Open-mindedness		0.229***	0.132***	0.127***
Negative emotionality		– 0.021	– 0.057	– 0.048
Conscientiousness		- 0.009	– 0.010	– 0.004
Agreeableness		0.039	– 0.023	– 0.020
Extraversion		0.071	0.007	0.012
Previous knowledge			0.455***	0.624***
Width of internet use				0.356**
Int: breadth of use*width of internet use				– 0.419**
F	46.650***	32.098***	62.963***	52.945***
R^2^	0.137	0.227	0.393	0.400
Adjusted R^2^	0.134	0.220	0.387	0.393
ΔR^2^	0.137	0.090	0.166	0.007

*p < 0.05; **p < 0.01; ***p < 0.001.

### The mediation effect of previous knowledge between open-mindedness and algorithmic awareness

In this study, we investigated the mediating effect of previous knowledge between open-mindedness and algorithmic awareness. Tables 4, 5 presented the relationship between these variables in the mediating effect. Table 4 demonstrated the direct effect of open-mindedness on algorithmic awareness and the indirect effect of open-mindedness on algorithmic awareness through the mediating effect of previous knowledge. Table 5 presented the specific indexes of the mediating effect’s total, direct and indirect effects.

**TABLE 4:** The mediation effect of previous knowledge between open-mindedness and algorithmic awareness.

Open-mindedness	Previous knowledge		Algorithmic awareness	
	*β*	Boot SE	*β*	Boot SE
Constant variables	1.2281***	0.2532	0.7003*	0.2693
Open-Mindedness	0.4508***	0.0472	0.1741***	0.0334
Previous knowledge			0.5231***	0.0588
Gender	0.1011	0.0537	0.0495	0.0388
Age	–0.0805**	0.0250	–0.0592**	0.0194
Education	0.1527***	0.0388	0.1359***	0.0292
R^2^	0.1735		0.3987	
F	46.1836***		83.0701 ***	

*p < 0.05; **p < 0.01; ***p < 0.001.

**TABLE 5:** Direct effects, indirect effects and overall effect.

	Effect	Boot SE	Boot LLCI	Boot ULCI
Overall effect	0.3494***	0.0361	0.2786	0.4203
Direct effect	0.1741***	0.0334	0.1065	0.2378
Mediation effect: low internet use	0.1961***	0.0260	0.1485	0.2490
Mediation effect: Medium internet use	0.1671***	0.0217	0.1270	0.2117
Mediation effect: High internet use	0.1357***	0.0220	0.0954	0.1809

*p < 0.05; **p < 0.01; ***p < 0.001.

Data analysis (as shown in Tables 4, 5) demonstrated that: open-mindedness significantly and positively predicted previous knowledge (*β* = 0.4508, *p* < 0.001); open-mindedness significantly and positively predicted algorithmic awareness (*β* = 0.1741, *p* < 0.001); previous knowledge significantly and positively predicted algorithmic awareness (*β* = 0.5231 *p* < 0.001); and open-mindedness indirectly and positively predicted algorithmic awareness through previous knowledge (*β* = 0.1719, *p* < 0.001). Table 5 presented the total, direct, and indirect effects of the mediated model, and the analysis indicated that effects above are within the 95% confidence interval, and the upper and lower bounds of the effects do not include 0, suggesting that the model is a mediating model. Thus, the second research question is answered as follows: there is a mediation effect of previous level of knowledge between the personality dimension of open-mindedness and algorithmic awareness.

The above results suggested that those with higher level of open-mindedness were more likely to be aware of the operations of algorithms in online platforms. Furthermore, level of open-mindedness indirectly affected level of algorithmic awareness through users’ amount of previous knowledge relating to algorithms. When users’ level of open-mindedness increased, they were more likely to obtain greater amount of algorithm related knowledge, thus increasing their level of awareness on the functions of algorithms in their daily life.

### The moderation effect of the breadth of internet use between previous knowledge and algorithmic awareness

Table 6 showed the moderation effect of the breadth of internet use between previous knowledge and algorithmic awareness. It was found that the breadth of internet use was significantly and positively predicted algorithmic awareness (*β* = 0.3404, *p* < 0.01) and then negatively moderated the correlation between previous knowledge and algorithmic awareness (*β* = –0.0886, *p* < 0.01); the mediating model index after adding the moderating variable was –0.0397 [SE = 0.0140, 95% CI = (-0.0675, –0.0134)]. Thus, research question 3 was answered: there is a statistically significant moderation effect of the breadth of internet use between previous knowledge and algorithmic awareness.

**TABLE 6:** The moderation effect of breadth of internet use between previous knowledge and algorithmic awareness.

IV	Algorithmic awareness	
	*β*	Boot SE
Constant	1.0747***	0.2575
Previous knowledge	0.5618***	0.0596
Width of internet use	0.3404**	0.1123
Int: breadth of use*width of internet use	– 0.0886**	0.0306
Gender	0.0587	0.0398
Age	– 0.0587**	0.0196
Education	0.1499***	0.0289
R^2^	0.3805	
F	89.8834***	

*p < 0.05; **p < 0.01; ***p < 0.001.

**FIGURE 2:**
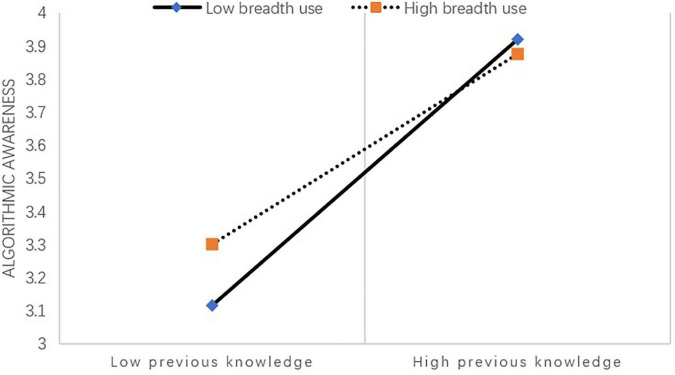
The moderation effect.

To further explore the moderation effect of internet use, Figure 2 demonstrated the changes in the influence between previous knowledge and algorithmic awareness moderated by different levels of the breadth of internet use. As shown in the figure, the slope increased from the high (M + 1SD) to low (M–1SD) breadth of internet use group. When the level of breadth of internet use is high, the positive relationship between previous knowledge and algorithmic awareness is significant (*β* = 0.3385, *t* = 9.8782, *p* < 0.001). The positive relationship between previous knowledge and algorithmic awareness was significant when the level of breadth of internet use was low, and the effect of previous knowledge on algorithmic awareness was more significant (*β* = 0.4732, *t* = 15.9778, *p* < 0.001).

**FIGURE 3:**
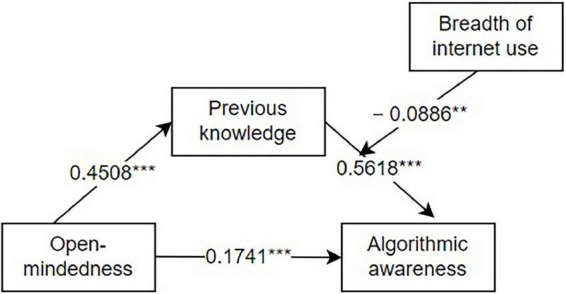
Model with coefficients. *p < 0.05; **p < 0.01; ***p < 0.001.

The results suggested that for respondents who use the internet with higher level of breadth, their level of algorithmic awareness increased with increased level of internet use breadth, while at the same time, the effect of previous knowledge on their increased level of algorithmic awareness is relatively weaker [SE = 0.0343, 95% CI = (0.2712, 0.4057)]. Whereas for participants with lower level of internet use breadth, the increase in amount of previous knowledge was correlated with a more significant increase in the level of algorithmic awareness [SE = 0.0296, 95% CI = (0.4151, 0.5313)]. Figure 3 presents the tested theoretical model and standardized regression coefficients.

## Conclusion and discussion

### Theoretical implications

In the pandemic era, it seems that there’s a mismatch between the increasing integration of algorithms in online platforms and users’ awareness of the operations of algorithms. In this study, we investigated the effect of personality traits on algorithmic awareness; more specifically, we examined the mediating role of previous knowledge regarding algorithms between open-mindedness and algorithmic awareness, as well as the moderating role of the breadth of internet use between previous knowledge and algorithmic awareness. Our results have demonstrated that among the five dimensions of Big Five personality traits, only open-mindedness is positively correlated with users’ awareness of algorithms; furthermore, previous knowledge plays a mediating role between open-mindedness and algorithmic awareness, and the breadth of internet use has a moderating effect between previous knowledge and algorithmic awareness. Our findings contribute to the literature on the personality traits and internet users’ algorithmic awareness in several ways.

First, our results indicate a limited effect of Big Five traits on users’ level of algorithmic awareness. In other words, among all the five dimensions of personality traits, only open-mindedness is the predictor of whether internet users are aware of algorithms’ usage and impact on their online content. This finding suggests that personality traits as a whole may not predominantly predict the gap in algorithmic awareness between internet users. But the positive correlation between the dimension of open-mindedness and algorithmic awareness is worth noticing. The more open-minded users are more likely to gain knowledge related to algorithms. As addressed earlier, users’ algorithm knowledge acquisition involves extant resources such as news reports, social networks, and direct interactions with algorithms through online engagement (Eslami et al., 2015; Rader and Gray, 2015; Klawitter and Hargittai, 2018; Cotter and Reisdorf, 2020). For individuals with a higher level of openness to experience, their willingness to try new things and engage in imaginative and creative activities seems to contribute to their accumulation of algorithmic knowledge through their experiences of online exploration. Furthermore, results of this study do not support the correlation between the other four dimensions of personality traits, including negative emotionality, conscientiousness, agreeableness, and extraversion, and algorithmic awareness. These four dimensions of personality traits, though have been shown to be related to issues like time spent online (Swickert et al., 2002; Landers and Lounsbury, 2006), internet addition (Kayiş et al., 2016), cyberbullying (Zhou et al., 2018), these amount of time and practices online do not necessarily relate to awareness of how algorithms operate. As addressed earlier, the awareness of algorithms, which are experience technologies (Blank and Dutton, 2012), requires active practices of exploring with curiosity than passively time spent online on algorithm-driven platforms (Eslami et al., 2015).

Second, this study emphasized the importance of previous knowledge in increasing the public’s awareness of algorithms in today’s algorithm-driven society. Results of this study indicate the mediating effect of users’ previous knowledge between open-mindedness and their level of algorithmic awareness. This finding is consistent with previous studies on the positive relationship between open-mindedness and the acceptance, trust, and adoption of new technologies (e.g., Huo et al., 2022). This suggests that people with a high level of openness would be more likely to try new things while using the internet. Furthermore, their rich experience accumulated through active engagement online would help to increase their awareness of the roles of algorithms in the platforms they frequently use. The results of several previous studies on algorithmic awareness, despite not paying attention to personality traits, have indicated that users’ level of engagement online is positively correlated with their awareness of the roles of algorithms in social media platforms such as Facebook (Rader and Gray, 2015). This finding seems to provide further implications for the current debates on improving users’ algorithmic awareness and furthering algorithmic literacy. In the research on this issue, early studies have emphasized users’ awareness of how a specific algorithm operates on specific media platforms such as Facebook or Google (Rader, 2014; Rader and Gray, 2015), whereas scholars from another line of research have proposed that users need to only have a general awareness of the use of algorithms in many domains of life (Hargittai et al., 2020). The results of the correlation between users’ mainly general and fragmented knowledge and their awareness of algorithms’ use and impact on a special domain of internet use (e.g., dynamics of online content) in this study suggest the integration of the above two lines of research. In other words, the increase in user knowledge of algorithms can help to deepen the understanding of the operations and impact of algorithms in users’ daily life.

Third, this study demonstrates the moderating effect of the breadth of internet use between previous knowledge and algorithmic awareness. That is, the breadth of engagement with online applications moderates the transition of users’ general algorithm knowledge into the specific awareness of how algorithms operate. This result is consistent with previous studies exploring the relationship between internet use, algorithmic knowledge accumulation and algorithmic awareness. For instance, for search engine users, the increase of frequency and bread of use contribute to greater level of knowledge of and understanding of how search engine works (Cotter and Reisdorf, 2020). Similarly, research conducted among social media users have also revealed that those having high usage frequency of Facebook (more than 20 times daily), are more likely to accumulate knowledge regarding algorithms; and the further exploration on this platform has increased their awareness of the functions and impact of news feed algorithms used by Facebook (Eslami et al., 2015).

### Practical implications

This study provides practical implications for improving people’s level of algorithmic awareness and increasing their autonomy in the new pandemic world. First of all, it is worth noticing that respondents in this study display a higher percentage of algorithmic awareness compared to those participants from many other studies. One possible explanation can be the societal features relating to AI in China. As the development of AI has become an essential step for realizing China’s dream of a cyber-superpower, both official narratives reflected on mainstream media and public discussions on social media surrounding AI have been similar, focusing on AI’s economic potential mainly from positive perspectives (Zeng et al., 2022). These positive evaluations of AI could to some extent increase the recognition of algorithms among the public. Another explanation could be the increase integration of AI in society that have sparked discussions about ethics relating to AI on social media platforms among scholars, journalists, IT industry actors, and the general public (Mao and Shi-Kupfer, 2021). In particular, since the start of the pandemic in China, the rapidly increasing news reports and public discussions on algorithm use distributed across popular social media have made the term ‘algorithm’ widely acknowledged among many internet users. For instance, in September 2020, an investigative report from China’s ‘People’ magazine entitled ‘Delivery drivers stuck in the system’ went viral on China’s popular social media platform WeChat within hours. This article elaborated how food delivery platforms utilize stringent algorithms to maximize profits at the cost of putting food deliverers and the public’s lives at risk. Moreover, this report caused a moment of national reckoning regarding the harm that algorithms present to people, partly because food delivery drivers are nearly omnipresent in Chinese cities. Through events such as these, members of the public in China, especially in cities, can accumulate knowledge on algorithms’ use and increase their awareness of algorithmic content production. Another explanation of the high level of awareness may be rated to the increasing number of users who have been using algorithm-driven online applications. According to a recent CNNIC report, by the end of June 2021, the number of China’s online video application users had reached 944 million (CNNIC, 2021). The most popular online video platforms in China, such as Douyin (TikTok), Kuaishou, and Bilibili, use algorithms to provide personalized content to their users. Thus, through their engagement on these platforms, users have accumulated knowledge relating to algorithms, which has led to their increased level of algorithmic awareness.

Second, in recent years, particularly since the start of the COVID-19 pandemic, widely adopted algorithm-driven applications have increased efficiency at both the individual and societal levels on the one hand but have also caused new concerns for both the government and the public on the other hand. Our findings have important policy implications for increasing the public’s algorithm awareness to enhance users’ autonomy in regard to encountering online content. In the context of China, ‘Internet information service algorithm recommendation management regulations’ were officially implemented on 1 March 2022. A noticeable included guideline is that users should be given the option to easily turn off algorithm recommendation services. Moreover, this guideline has the potential to shape the global landscape of algorithm regulation. Recently, many online platforms that are widely used in the domains of online shopping, entertainment, etc., have started providing options for users to turn off the algorithms used by their platforms following this regulation. This change is especially important for individuals living in an algorithm-driven world, as they can have a relatively high level of autonomy in deciding what content they encounter online and make decisions about the information they gather based on their own choices. However, the precondition for users to turn off the algorithms is that they are aware of the algorithms used in the platforms and the impact that these algorithms have on their daily life. Therefore, increasing users’ algorithm awareness is the crucial first step for policy-makers and organizations who aim to improve users’ online autonomy.

### Limitations of the study

There are several limitations of the present study. First, our sample tended to be drawn from well-educated, young and middle-aged groups. On the one hand, the characteristics shared in these groups of respondents helped to guarantee the quality of the personality data collected through a 60-question scale. However, on the other hand, the level of algorithmic awareness of those drawn from the less-educated and older age groups was less likely to be represented in our sample. Considering that many of these individuals are also users who are influenced by algorithms, in the future, we need to conduct studies among these groups using methods suitable for them to gain an awareness of algorithms. Second, the measures of algorithmic awareness and the Big Five personality traits were based on respondents’ self-reports. We recommend that future studies either use different methods or conduct studies among different groups to better replicate our findings. Third, although the adapted Algorithmic Media Content Awareness Scale (AMCA-scale) has been tested on three different online platforms, namely, Facebook, Netflix, and YouTube, among U.S. respondents (Zarouali et al., 2021), there has been a lack of validation of this scale on platforms outside the Western context. Therefore, future studies in this field should be conducted to further verify the generalizability of this scale using samples of online platforms used in different countries.

## Data availability statement

The raw data supporting the conclusions of this article will be made available by the authors, without undue reservation.

## Ethics statement

Ethical review and approval was not required for the study on human participants in accordance with the local legislation and institutional requirements. Written informed consent for participation was not required for this study in accordance with the national legislation and the institutional requirements.

## Author contributions

WF contributed to the research idea, literature review, and data analysis and model. JJ contributed to the analysis with constructive discussions of the study. Both authors contributed to the article and approved the submitted version.
