# Supplementary material for: Unpacking the effects of personality traits on algorithmic awareness: The mediating role of previous knowledge and moderating role of internet use
Source: Front Psychol. 2022 Sep 6;13:953892. doi: 10.3389/fpsyg.2022.953892 (PMC9485722; doi:10.3389/fpsyg.2022.953892)
Supplement: Supplementary file 1 [file Data_Sheet_1.docx]

APPENDIX

(1) What is your gender

• female

• male

(2) What is your age

• 10-19

• 20-29

• 30-39

• 40-49

• 50-59

• 60 and above

(3) What is your level of education

| • Primary |
| --- |
| • Junior secondary |
| • Senior secondary |
| • Vocational college |
| • Undergraduate and above |

(4) Number of frequently used online applications

• Below 10

• 10-19

• 20-29

• 30-39

• 40 and above

(5) Algorithms have been integrated in many online platforms to arrange the online content we encounter, e.g., news on *Jinritoutiao*, videos on *douyin* and *kuaishou*, searching results on *baidu*. Please rate your level of knowledge relating to algorithms:

• Completely don’t know

• Rarely know

• Know a little

• Know a lot

• Completely know

*Please give us your opinion on your level of awareness on the operations of algorithms in online applications (e.g., Jinritoutiao, Douyin, Taobao, Baidu), in terms of your rating for the following statements:*

(6) “Algorithms are used to recommend content to me”

• Completely don’t know

• Rarely know

• Know a little

• Know a lot

• Completely know

(7) “Algorithms are used to prioritize certain content above others”

• Completely don’t know

• Rarely know

• Know a little

• Know a lot

• Completely know

(8) “Algorithms are used to tailor certain content to me”

• Completely don’t know

• Rarely know

• Know a little

• Know a lot

• Completely know

(9) “Algorithms are used to show someone else see different content than I get to see”

• Completely don’t know

• Rarely know

• Know a little

• Know a lot

• Completely know

(10) “Algorithms are used to show me content online based on automated decisions”

• Completely don’t know

• Rarely know

• Know a little

• Know a lot

• Completely know

(11) “Algorithms do not require human judgments in deciding which content to show me”

• Completely don’t know

• Rarely know

• Know a little

• Know a lot

• Completely know

(12) “Algorithms make automated decisions on what content I get to see”

• Completely don’t know

• Rarely know

• Know a little

• Know a lot

• Completely know

(13) “The content that algorithms recommend to me on certain platform depend on my online behavior on that platform”

• Completely don’t know

• Rarely know

• Know a little

• Know a lot

• Completely know

(14) “The content that algorithms recommend to me online depend on my online behavioral data”

• Completely don’t know

• Rarely know

• Know a little

• Know a lot

• Completely know

(15) “The content that algorithms recommend to me depend on the data that I make available online”

• Completely don’t know

• Rarely know

• Know a little

• Know a lot

• Completely know

(16) “It is not always transparent why algorithms decide to show me certain content”

• Completely don’t know

• Rarely know

• Know a little

• Know a lot

• Completely know

(17) “The content that algorithms recommend to me can be subjected to human biases such as prejudices and stereotypes”

• Completely don’t know

• Rarely know

• Know a little

• Know a lot

• Completely know

(18) “Algorithms use my personal data to recommend certain content, and this has consequences for my online privacy

• Completely don’t know

• Rarely know

• Know a little

• Know a lot

• Completely know

(19) If the following statements are descriptions about yourself, please rate your level of agreement on a scale of 1–5, with 1 being “disagree strongly” and 5 being “agree strongly”:

| 1  Disagree  strongly | 2  Disagree  a little | 3  Neutral;  no opinion | 4  Agree  a little | 5  Agree  strongly |
| --- | --- | --- | --- | --- |
| **I am someone who . . .**  1. Is outgoing, sociable.  2. Is compassionate, has a soft heart.  3. Tends to be disorganized.  4. Is relaxed, handles stress well.  5. Has few artistic interests.  6. Has an assertive personality.  7. Is respectful, treats others with respect.  8. Tends to be lazy.  9. Stays optimistic after experiencing a setback.  10. Is curious about many different things.  11. Rarely feels excited or eager.  12. Tends to find fault with others.  13. Is dependable, steady.  14. Is moody, has up and down mood swings.  15. Is inventive, finds clever ways to do things.  16. Tends to be quiet.  17. Feels little sympathy for others.  18. Is systematic, likes to keep things in order.  19. Can be tense.  20. Is fascinated by art, music, or literature.  21. Is dominant, acts as a leader.  22. Starts arguments with others.  23. Has difficulty getting started on tasks.  24. Feels secure, comfortable with self.  25. Avoids intellectual, philosophical discussions.  26. Is less active than other people.  27. Has a forgiving nature.  28. Can be somewhat careless.  29. Is emotionally stable, not easily upset.  30. Has little creativity.  31. Is sometimes shy, introverted.  32. Is helpful and unselfish with others.  33. Keeps things neat and tidy.  34. Worries a lot.  35. Values art and beauty.  36. Finds it hard to influence people.  37. Is sometimes rude to others.  38. Is efficient, gets things done.  39. Often feels sad.  40. Is complex, a deep thinker.  41. Is full of energy.  42. Is suspicious of others’ intentions.  43. Is reliable, can always be counted on.  44. Keeps their emotions under control.  45. Has difficulty imagining things.  46. Is talkative.  47. Can be cold and uncaring.  48. Leaves a mess, doesn’t clean up.  49. Rarely feels anxious or afraid.  50. Thinks poetry and plays are boring.  51. Prefers to have others take charge.  52. Is polite, courteous to others.  53. Is persistent, works until the task is finished.  54. Tends to feel depressed, blue.  55. Has little interest in abstract ideas.  56. Shows a lot of enthusiasm.  57. Assumes the best about people.  58. Sometimes behaves irresponsibly.  59. Is temperamental, gets emotional easily.  60. Is original, comes up with new ideas. | | | | |
